# COVID-19 and the emerging research trends in environmental studies: a bibliometric evaluation

**DOI:** 10.1007/s11356-021-13098-z

**Published:** 2021-02-24

**Authors:** Muhammad Usman, Yuh-Shan Ho

**Affiliations:** 1 PEIE Research Chair for the Development of Industrial Estates and Free Zones, Center for Environmental Studies and Research, Sultan Qaboos University, Al-Khoud, 123 Muscat, Oman; 2 Trend Research Centre, Asia University, No. 500, Lioufeng Road, Wufeng, Taichung, 41354 Taiwan

**Keywords:** COVID-19, SARS-CoV-2, Environment, Air, Water, Bibliometrics

## Abstract

The ongoing pandemic of the coronavirus disease 2019 (COVID-19) is a global health emergency. Thousands of articles have been published to tackle this crisis. Here, a bibliometric study of the publications in environmental studies has been conducted to identify the emerging research trends in this field in the era of COVID-19. Bibliometric analysis serves as a useful tool to evaluate research productivity and scholarly trends in a field. For this, publications were searched in nine environment-related subject categories indexed in Science Citation Index Expanded (SCI-EXPANDED) database of the Web of Science Core Collection. A bibliometric evaluation of 495 relevant documents was performed to identify various essential research indicators, including the type of the publication, the most prominent journals, subject categories, authors, institutions, and the countries, that contributed significantly to this theme. Major focus of this bibliometric study is to illustrate the potential research hotspots emerged during this pandemic. It has been found that significant amount of research has been conducted for the assessment of environmental quality and its contribution in environmental transmission of COVID-19. In addition to its positive impacts on environment, COVID-19 has contributed indirectly in worsening many environmental threats such as increased exposure to disinfectants and antimicrobials, poor solid waste management, and food insecurity. Researchers have also been focusing on the strategies for the planning of post-COVID-19 cities and buildings and to protect the ecology. This bibliometric study allowed the visualization of research agenda in the field of environmental studies during this pandemic.

## Introduction

The ongoing pandemic of the coronavirus disease 2019 (COVID-19) is a global health emergency. This disease has infected more than 68 million people causing more than 1.5 million deaths worldwide (Worldometers 2020). Governments are responding at local, national, regional, and global levels in the face of an evolving body of evidence and other circumstances. For that, authorities and scientific community are seeking for emergent insights on how to tackle this crisis. As expected, major portion of the research is related to the medical interventions for the prevention and treatment of this disease. However, environmental community has contributed significantly in research related the implications of this pandemic in environment. In addition to the research reported in regular issues, many journals have devoted special issues to cover the research on this theme.

Present study is intended to evaluate the emerging research trends with the help a bibliometric analysis of the existing literature on COVID-19. Bibliometrics is particularly useful in facilitating the researchers to examine, interpret, and derive indicators on the progress and dynamics of scientific knowledge on a subject. Bibliometric analysis of the existing literature on a particular theme allows recognition of the future research directions and facilitates the decision making by decreasing the margin of error (Vanzetto and Thomé 2019; Colares et al. 2020). Therefore, present study was performed to evaluate the research related to COVID-19 that appeared in environment-related journals categorized in SCI-EXPANDED. This database was chosen as its comprehensive coverage of the multidisciplinary journals makes it the most efficient database to represent the search results. There are 254 subject categories in SCI-EXPANDED and each journal is assigned to at least one category. For this study, nine categories were selected due to their relevance to the environment including (i) environmental sciences; (ii) environmental engineering; (iii) public, environmental, and occupational health; (iv) ecology; (v) water resources; (vi) green and sustainable science and technology; (vii) meteorology and atmospheric sciences; (viii) soil science; and (ix) forestry. A SCI-EXPANDED-based analysis of the search result was performed to identify the important research hotspots along with other essential indicators like the type of publications and the most prominent authors, scientific journals, institutions, and the countries that have contributed substantially to this subject. This bibliometric study would facilitate the researchers to identify the potential research hotspots and latest trends in this field.

## Methodology

For this study, the Clarivate Analytics Web of Science Core Collation was used which is an online version of the Science Citation Index Expanded (SCI-EXPANDED). The SCI-EXPANDED Web of Science database has been chosen because of its high efficiency to represent search results. Therefore, its use has been recommended to search for journals and references (Vanzetto and Thomé 2019). These authors reported that WoS was more efficient at representing the results, providing 61.5% more articles than the Scopus database. Though it is highly efficient database at representing the search results, its search representation could vary as compared to other databases like Scopus, Google Scholar. This limitation merits to be acknowledged. It should also be noted that research on some themes is not conclusive and considering the evolving nature of this pandemic, more reviews on this topic would be required. It is worth mentioning that certain impacts (e.g., socio-economic impacts) take some more time to appear (Sharifi and Khavarian-Garmsir 2020).

On the basis of a preliminary review (data not shown), data was retrieved on September 14, 2020 by using the following keywords within title (TI), abstract (AB), and author keywords (AK) in advanced search: “COVID-19” or “COVID-2019” or “2019-nCoV” or “COVID19” or “Coronavirus Disease 2019” or “severe acute respiratory syndrome coronavirus 2” or “SARS-CoV-2.” Use of quotation marks (“ ”) allows finding the exact search term by avoiding the synonym and lemmatization features of Web of Science which are by default ON in search settings of this database. However, to find exact expressions, we relied on the use of Boolean operator “or” to ensure that at least one search term appeared.

These keywords were searched in the nine Web of Science categories related to environment including environmental sciences (265 journals), environmental engineering (53 journals), public, environmental and occupational health (193 journals), ecology (168 journals), water resources (94 journals), green and sustainable science and technology (41 journals), meteorology and atmospheric sciences (93 journals), soil science (38 journals), and forestry (68 journals). This search yielded 2217 documents which were then screened by relying on the title and or abstract to eliminate the articles which were irrelevant to the theme, e.g., medical interventions to address COVID-19, studies on the impacts of COVID-19 on public health and behavior. After this initial screening, 570 documents were shortlisted from which early access (which do not have the publication information such as publication year, volume or page numbers) were excluded. This led to a total of 495 publications which has been used for further screening and analysis.

Impact factors of journals reported in present article (IF_2019_) are based on the latest Journal Citation Reports 2019. The obtained records were rearranged with Microsoft Excel 2016 as explained previously (Li and Ho 2008). It is worth mentioning that the term “corresponding author” has been retained which is designated as the “reprint author” in the SCI-EXPANDED database (Chiu and Ho 2007). In articles having multiple corresponding authors, only the last corresponding author has been retained. Affiliations of authors in England, Scotland, Northern Ireland, and Wales were categorized as being from the United Kingdom (UK) (Chiu and Ho 2005).

## Results and discussion

### Characteristics of the documents published on this theme

Firstly, publications were categorized into different types indexed in the Web of Science (Table 1). Among them, article category in document types is ranked 1^st^ (62% of 495 publications) followed by editorial material (20%), letter (9.3%), review (7.9%), news item (0.61%), correction (0.40%), and book review (0.20%). A total of 99 editorial materials were published in 62 journals. A total of 39 reviews appeared in 18 journals in which *Science of the Total Environment* issued the maximum number, 11, of meeting abstracts (28% of 39 reviews).

**Table 1:** Authors according to document type

Document type	TP	%	AU	APP
Article	305	62	1500	4.9
Editorial material	99	20	420	4.2
Letter	46	9.3	166	3.6
Review	39	7.9	184	4.7
News item	3	0.61	3	1.0
Correction	2	0.40	5	2.5
Book review	1	0.20	1	1.0

*TP*, number of articles; *AU*, number of authors; *APP*, number of authors per publication

The Web of Science denotes article as “reports of research on original works. Includes research papers, features, brief communications, case reports, technical notes, chronology, and full papers that were published in a journal and/or presented at a symposium or conference.” Therefore, article category (composed of 305 articles) was chosen for further analysis to depict the correct picture of emerging research landscape in environment due to COVID-19.

### Web of Science categories and journals

Web of Science category of environmental sciences published the most of COVID-19 articles with 242 (79% of 305 articles), followed by public, environmental, and occupational health (81 articles; 27%); green and sustainable science and technology (35; 11%); environmental engineering (12; 3.9%); meteorology and atmospheric sciences (11; 3.6%); ecology (9; 3.0%); water resources (8; 2.6%); soil science (1; 0.33%); and forestry (1; 0.33%). It has been pointed out that WOS categories the journals in multiple categories. For example, *Water Research* is categories in environmental engineering, environmental sciences, and water resources that leads to the sum of percentages >100% (Usman and Ho 2020).

In total, 305 COVID-19 articles were published in 71 journals in nine environment-related categories. The top 15 most productive journals with their IF_2019_ and number of authors per publication (APP) are listed in Table 2. *Science of the Total Environment* (IF_2019_ = 6.551), categorized in environmental sciences, published the most articles (100 articles; 33% of 305 articles) followed distantly by other journals. Considering journal’s impact factor, *Nature Climate Change* ranked top in category of meteorology and atmospheric sciences (1^st^ of 93 journals), stood first with the highest IF_2019_ of 20.893 (one article), followed by *MMWR-Morbidity and Mortality Weekly Report* (IF_2019_ = 13.606, five articles), *Nature Ecology & Evolution* (IF_2019_ = 12.541, two articles), *Renewable & Sustainable Energy Reviews* (IF_2019_ = 12.110, one article). Comparison of the top 15 productive journals (Table 2) indicates that articles published in *MMWR-Morbidity and Mortality Weekly Report* had the highest *APP* of 19 followed by those in *Environment International* (APP = 14).

**Table 2:** Top 15 journals (TP ≥ 3)

Journal	TP (%)	IF_2019_	AU	APP
Science of the Total Environment	100 (33)	6.551	518	5.2
Sustainability	31 (10)	2.576	128	4.1
International Journal of Environmental Research and Public Health	29 (10)	2.849	116	4.0
Aerosol and Air Quality Research	24 (7.9)	2.735	136	5.7
American Journal of Infection Control	8 (2.6)	2.294	40	5.0
Air Quality Atmosphere and Health	7 (2.3)	2.870	31	4.4
Environmental Research	6 (2.0)	5.715	24	4.0
Epidemiology and Infection	6 (2.0)	2.152	27	4.5
MMWR-Morbidity and Mortality Weekly Report	5 (1.6)	13.606	97	19
Bulletin of Environmental Contamination and Toxicology	4 (1.3)	1.657	18	4.5
Frontiers in Public Health	4 (1.3)	2.483	22	5.5
Journal of Hospital Infection	4 (1.3)	3.271	17	4.3
Environment International	3 (1.0)	7.577	41	14
Environmental Science & Technology Letters	3 (1.0)	7.678	20	6.7
Journal of Urban Health-Bulletin of the New York Academy of Medicine	3 (1.0)	2.356	6	2.0

*TP*, number of total articles; *IF*_2019_, journal impact factor in 2019; *AU*, number of authors; *APP*, number of authors per article (*AU*/*TP*)

### Countries that have published the most in this field

The contribution of countries relied on the affiliation of at least one author of articles. Authors from 61 countries contributed in these 304 articles published in this theme. Among these 304 articles, there were 196 country-independent publications (64% of 304 articles) from 41 countries and 108 internationally collaborative publications (36% of 304 articles) from 53 countries. In this context, countries’ research performance was evaluated on the basis of six publication indicators including total (TP), country independent (IP), internationally collaborative (CP), first author (FP), corresponding author (RP), and single author (SP) articles (Hsu and Ho 2014; Usman and Ho 2020). Table 3 shows the top 15 productive countries which include six Asian countries, five European countries, three American countries, and Oceania country. There were no African countries in the top 15. The most productive African country was South Africa with three articles ranked 29th. All of the seven major industrialized countries of the world (G7) including the USA, Italy, the UK, Japan, Canada, France, and Germany were ranked in the top 15. China published the most articles with a TP of 76 articles (25% of 304 articles), a CP of 40 articles (37% of 108 internationally collaborative articles), an FP of 65 articles (21% of 304 first author articles), while the USA ranked top with an IP of 41 articles (21% of 196 country independent articles), an RP of 54 articles (18% of 304 corresponding author articles) and an SP of 10 articles (24% of 42 single author articles).

**Table 3:** Top 15 countries (TP ≥ 8)

Country	Rank (TP)	Rank (IP)	Rank (CP)	Rank (FP)	Rank (RP)	Rank (SP)
China	1 (76)	2 (36)	1 (40)	1 (65)	2 (53)	6 (1)
USA	2 (71)	1 (41)	2 (30)	2 (50)	1 (54)	1 (10)
Italy	3 (35)	4 (20)	4 (15)	3 (29)	3 (28)	3 (4)
India	4 (32)	3 (21)	6 (11)	4 (28)	4 (24)	2 (6)
UK	5 (21)	8 (4)	3 (17)	8 (7)	9 (7)	4 (2)
Australia	6 (15)	20 (1)	5 (14)	8 (7)	9 (7)	6 (1)
Brazil	6 (15)	5 (11)	19 (4)	5 (13)	5 (15)	N/A
Japan	8 (14)	8 (4)	7 (10)	7 (8)	7 (9)	N/A
Spain	9 (13)	6 (6)	9 (7)	6 (9)	7 (9)	4 (2)
Taiwan	10 (11)	7 (5)	12 (6)	10 (5)	6 (10)	N/A
Canada	11 (10)	20 (1)	8 (9)	18 (3)	35 (1)	6 (1)
France	12 (9)	14 (2)	9 (7)	10 (5)	16 (4)	6 (1)
Germany	12 (9)	12 (3)	12 (6)	10 (5)	11 (5)	6 (1)
Pakistan	14 (8)	14 (2)	12 (6)	24 (2)	11 (5)	N/A
Saudi Arabia	14 (8)	20 (1)	9 (7)	31 (1)	24 (2)	6 (1)

*TP*, total number of articles; *IP*, number of country-independent articles; *CP*, number of internationally collaborative articles; *FP*, number of first author articles; *RP*, number of corresponding author articles; *SP*, number of single author articles; N/A, not available

### Institutions that have published the most on COVID-19 articles

For this, six publication indicators (Table 4) were used which are derived as explained elsewhere (Hsu and Ho 2014). Among the total 304 articles, there were 29 single institution articles (30%), whereas inter-institutional collaborations resulted in 212 articles (70%). The top 13 institutions with four articles or more are shown in Table 4. Among them, seven are located in China, two in Taiwan, and one in each the UK, Italy, and Saudi Arabia. The Chinese Academy of Sciences in China is the most prominent institute having a TP of seven articles (2.3% of 304 articles), a CP of seven articles (3.3% of 212 internationally collaborative articles), and an RP of three articles (0.98% of 304 corresponding author articles). The Centers for Disease Control and Prevention (CDC) in the USA, the Chapman University in the USA, the University of Sao Paulo in Brazil, and the University of Wah in Pakistan also published three corresponding author articles. The University of Calabria in Italy ranked top in institute independent articles with an IP of two articles (0.66% of 92 institute independent articles). The Karunya Institute of Technology and Sciences in India, the National Institute of Research and Development for Optoelectronics in Romania, the Purdue University in the USA, and the University of Naples Federico II in Italy also published two institute independent articles. Hefei University of Technology in China ranked top in first author articles with an FP of four articles (1.3% of 304 first author articles). University Exeter in the UK was the only top 13 institutes published single author articles. The Karunya Institute of Technology and Sciences in India was the only institute published two single author articles. It is worth mentioning that the Chinese Academy of Sciences has multiple branches in different cities (Li et al. 2009), whereas its publications were pooled as of one organization at present. However, different rankings could be yielded if publications were divided according to its branches (Li et al. 2009). COVID-19 articles published in environment-related categories were published by Chinese Academy of Sciences in Beijing, Xian, Lanzhou, and Guangzhou, respectively.

**Table 4:** Top 13 institutions (TP ≥ 4)

Institute	Rank (TP)	Rank (IP)	Rank (CP)	Rank (FP)	Rank (RP)	Rank (SP)
Chinese Academy of Sciences, China	1 (7)	N/A	1 (7)	8 (2)	1 (3)	N/A
Peking University, China	2 (6)	6 (1)	2 (5)	8 (2)	6 (2)	N/A
Shandong University, China	2 (6)	6 (1)	2 (5)	2 (3)	34 (1)	N/A
King Saud University, Saudi Arabia	4 (5)	N/A	2 (5)	N/A	N/A	N/A
Lanzhou University, China	4 (5)	6 (1)	5 (4)	2 (3)	6 (2)	N/A
China Medical University, Taiwan	6 (4)	N/A	5 (4)	36 (1)	6 (2)	N/A
Chinese University of Hong Kong, China	6 (4)	N/A	5 (4)	8 (2)	6 (2)	N/A
Fudan University, China	6 (4)	N/A	5 (4)	8 (2)	6 (2)	N/A
Hefei University of Technology, China	6 (4)	N/A	5 (4)	1 (4)	N/A	N/A
Hunan University of Arts and Science, China	6 (4)	N/A	5 (4)	36 (1)	N/A	N/A
National Central University, Taiwan	6 (4)	6 (1)	12 (3)	36 (1)	34 (1)	N/A
University of Calabria, Italy	6 (4)	1 (2)	38 (2)	2 (3)	6 (2)	N/A
University of Exeter, UK	6 (4)	N/A	5 (4)	8 (2)	34 (1)	2 (1)

*TP*, total number of articles; *IP*, number of institute independent articles; *CP*, number of inter-institutionally collaborative articles; *FP*, number of first author articles; *RP*, number of corresponding author articles; *SP*, number of single author articles; N/A, not available

### Research hotspots and their trends

#### Air quality assessment and its significance

**Fig. 1:**
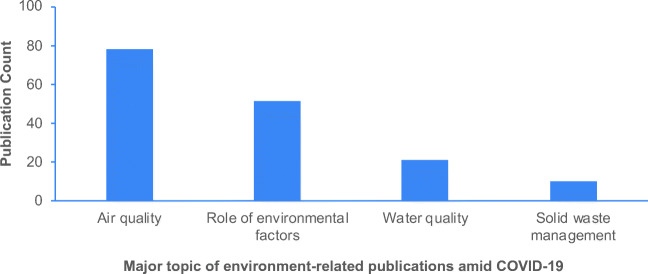
Major topics and number of environment-related publications in the era of COVID-19

Air quality assessment has been the major focus of COVID-19-related research in environmental science that has been the focus of at least 78 articles (Fig. 1). This trend is also evident from the frequent distribution of the air associated words in article titles, author keywords, abstract, and *KeyWords Plus* (Table 5). This distribution of words provides the most important information that authors of a manuscript want to convey to their readers (Wang and Ho 2016).

**Table 5:** Top 20 most used words

Rank	Words in title	*TP*	Author keywords	TP	Words in Abstract	TP	KeyWords Plus	TP
1	COVID-19	212	COVID-19	158	COVID-19	218	Transmission	21
2	Air	58	SARS-COV-2	42	2020	144	Pollution	20
3	Pandemic	47	Coronavirus	37	Pandemic	115	Coronavirus	19
4	SARS-COV-2	42	Air pollution	25	Coronavirus	102	Impact	17
5	Quality	40	Lockdown	25	Air	99	Outbreak	16
6	Impact	38	Air quality	15	Disease	93	Health	14
7	Lockdown	36	NO_2_	15	Health	85	PM_2.5_	13
8	China	35	Pandemic	15	Spread	82	Air pollution	12
9	Transmission	25	Temperature	12	2019	78	Influenza	12
10	Outbreak	22	PM_2.5_	10	Cases	70	Mortality	11
11	Coronavirus	21	India	8	Control	70	China	10
12	Case	20	PM_10_	8	Virus	70	COVID-19	10
13	Pollution	20	Transmission	8	Quality	65	Risk	9
14	Health	19	Humidity	7	SARS-COV-2	64	Exposure	8
15	India	18	O-3	7	Significant	63	Model	8
16	Environmental	16	SO_2_	7	World	62	Quality	8
17	Food	15	Airborne transmission	6	March	61	Temperature	8
18	Changes	14	CO	6	Outbreak	61	Virus	8
19	Temperature	14	Sustainability	6	Lockdown	60	Acute respiratory syndrome	7
20	Environment	13	AQI	5	Infection	59	Air quality	7

COVID-19 pandemic has been suggested as a blessing in disguise in reducing the environmental pollution (Muhammad et al. 2020). Reduction in noise pollution has been recorded amid lockdown in Shillong, India (Somala 2020). Impacts of lockdown on the emissions of CO_2_, CO, NO_2_, O_3_, PM_2.5_, PM_10_, and SO_2_ have been assessed at local as well as international scale. Significant reductions in the emissions of CO_2_, CO, NO_2_, and SO_2_ have been noted due to the lockdown measures in many cities around the world. It has been reported that lockdown measures have pronounced impact in reducing the concentration of pollutants (like CO, NO_x_) which are directly related to the transportation sector (Sharifi and Khavarian-Garmsir 2020). However, decrease in the concentration of PM was less significant because it is mainly contributed by non-transportation sources such as biomass burning, industrial emissions, or residential heating (Sharifi and Khavarian-Garmsir 2020). Therefore, policy measures to address air pollution should focus on both transportation and non-transportation sectors (e.g., residential sector). For example, Sharma et al. (2020) analyzed the effects of lockdown on air quality in 22 cities of India. They noted a decline of 43%, 31%, 10%, and 18% in PM_2.5_, PM_10_, CO, and NO_2_, respectively while 17% increase in the concentration of ozone (O_3_). It should be noted that reductions in NO_x_ could create an imbalance in air pollution that could change the air pollution chemistry. For example, unprecedented decline in NO_x_ can increase in the concentration of O_3_ due to lower titration of O_3_ by NO (Sicard et al. 2020). Similar increase in O_3_ concentration accompanied by a decline in the contents of NO_2_ and other pollutants (PM_2.5_, PM_10_, CO) have been noted in Wuhan (by 145%) (Wang et al. 2020b) and Milan (by a factor of 2.25) (Zoran et al. 2020a). Comparison of air quality in four European cities (Nice, Rome, Valencia, and Turin) and one Chinese city (Wuhan) revealed a substantial decline in NOx (~ 56%) in all cities (Sicard et al. 2020). Reductions in PM were much lower in European cities (< 10%) than in Wuhan (42%). Moreover, concentration of O_3_ increased in all cities (36% in Wuhan and 17% in Europe). These studies highlight the challenge of formation of secondary pollutants that should also be considered in addition to the efforts to reduce the primary pollutant emissions. For this, better understanding of complex chemistry of air pollutant formation, transport, and temporal variability is required. Moreover, further research is needed to understand the role of meteorological conditions in dictating the concentration of air pollutants. For example, a modeling study in China associated the occurrence of severe air pollution episodes despite reduced activities to the unfavorable meteorology (Wang et al. 2020c). Therefore, role of meteorology should also be considered in designing emission control strategies. Significant associations of air pollution in exacerbating COVID-19 have also been reported which are discussed in section “Environmental factors affecting the transmission of SARS-CoV-2 and disease vectors.”

#### Water quality assessment and its significance in COVID-19

Access to clean water plays a crucial role in ensuring adequate health conditions for people and controlling the spread of this virus particularly via handwashing. Therefore, water demand has been found to increase during this pandemic (Balacco et al. 2020). Researchers have also been investigating the impact of this pandemic on the quality of the water resources. Overall an improvement in the quality of the surface water was witnessed in the reports from Italy (Braga et al. 2020), India (Mandal and Pal 2020; Yunus et al. 2020) etc. Similarly, groundwater quality has also been improved (Selvam et al. 2020) due to a decrease in agricultural and industrial activities. It is worth mentioning that studies on the assessment of the water quality are quite limited as compared to those on the air quality.

Due to the fecal shedding of the SARS-CoV-2 from COVID-19 infected persons, use of the wastewater-based epidemiology has received significant attention among the scientific community. Since its first detection in sewage in the Netherlands (Lodder and Husman 2020), SARS-CoV-2 has been identified in sewage in many countries such as Australia (Ahmed et al. 2020a), Czech Republic (Mlejnkova et al. 2020), Italy (La Rosa et al. 2020), Japan (Haramoto et al. 2020), and Spain (Randazzo et al. 2020) where it correlated well with the clinically confirmed patients. People excrete this virus well before they develop symptoms for this disease. Therefore, wastewater-based which could enable the quick detection of this virus before the clinical confirmations. It has been suggested by Hart and Halden (2020) on the basis of a computational analysis that theoretically; it is possible to detect one infected individual among 100 to 2,000,000 persons. However, collection and interpretation of data for wastewater surveillance is an emerging field. This becomes particularly important to choose the correct virus concentration method (Ahmed et al. 2020b).

Despite its benefits in wastewater-based epidemiology, the existence of SARS-CoV-2 in wastewater can have potential health risks. Although there is currently no evidence of fecal-oral transmission of SARS-CoV-2, it remains a significant possibility particularly in developing communities with weak water and sewage infrastructure (Usman et al. 2020b). Similarly, researchers have also raised concerns about environmental transmission of SARS-CoV-2 in recreational water (Cahill and Morris 2020) and rehabilitation pools (Romano-Bertrand et al. 2020). Moreover, McDermott et al. (2020) suggested that role of virus-laden bioaerosols released from the toilets in transmission of this virus must not be ignored. Therefore, standard precautions, wastewater disinfection and raising awareness are called for. Further insights into the disinfection are provided in the Section “Increased exposure to the emerging environmental pollutants and disinfectants”

#### Environmental factors affecting the transmission of SARS-CoV-2 and disease vectors

Transmission of SARS-CoV-2 mainly occurs through inhalation of respiratory droplets or through contact with contaminated surfaces (WHO 2020). As recognized by the WHO, airborne transmission can occur during medical procedures that generate aerosols. (WHO 2020). Moreover, WHO is also evaluating the airborne transmission of this virus through virus-laden particles in the absence of aerosol-generating medical procedures (WHO 2020). Therefore, environmental researchers have also been focusing on evaluating the persistence and transmission of this virus in the environment and the environmental factors affecting its fate. As detailed in above sections, researchers have been raising concerns for the environmental transmission of this virus through air (Morawska and Cao 2020; Wang and Yoneda 2020) and wastewater (Cahill and Morris 2020; Romano-Bertrand et al. 2020; Usman et al. 2020b). Transmission dynamics of this virus can be affected by the characteristics of the ambient environment. Therefore, researchers have been studying the effect of different environmental and meteorological parameters such as humidity, temperature, wind speed, pollution levels, and indoor ventilation.

Regarding the impact of ambient temperature on the spread of COVID-19, researchers reported conflicting results. For example, Jahangiri et al. (2020) found that COVID-19 transmission rate exhibited low sensibility towards temperature in a study of 31 provinces across Iran and ruled out the reasoning that COVID-19 cases are less in warmer climates as compared to that of moderate or cold regions. However, Lin et al. (2020) found a negative and exponential relationship between temperature and COVID-19 transmission rate in a study of 20 provinces/municipalities across China. Therefore, authors suggested that it would be challenging to mitigate COVID-19 spread in cold regions (Lin et al. 2020). Similarly, Prata et al. (2020) reported in their study of 27 cities of Brazil that at average temperature below 25.8 °C, each 1 °C increase was linked to a − 4.8951% decline in the decrease in the number of daily cumulative confirmed COVID-19 cases. However, authors did not find any evidence to support that COVID-19 cases could decrease when the weather becomes warmer, above 25.8 °C (Prata et al. 2020). A negative correlation between temperature and COVID-19 cases has also been reported by Sobral et al. (2020). They found that each 1 °F increase in average daily temperature was associated to a decline of 6.4 cases/day. However, they noted a positive correlation between precipitation and COVID-19 transmission (Sobral et al. 2020). A study across 33 locations in China reported that association of air quality index (AQI) and COVID-19 cases is statistically significant. The impact of AQI is pronounced in the temperature range of 10 °C ≤ T < 20 °C and in the relative humidity (RH) range of 10% ≤ RH < 20% (Xu et al. 2020). The transmission of the virus may be facilitated by the drier air (Sharifi and Khavarian-Garmsir 2020; Xu et al. 2020), whereas in humid air, airborne transmission of virus droplets is decreased as they are more likely to drop down (Sharifi and Khavarian-Garmsir 2020). Similar findings have been reported by Pani et al. (2020) who found a positive significant association of temperature, dew point, and humidity with virus transmission. Regarding wind speed, SARS-CoV-2 transmission is negatively correlated with wind speed in Italy (Zoran et al. 2020b), Iran (Ahmadi et al. 2020) and Singapore (Pani et al. 2020). Accumulation of air pollutants is more likely to be pronounced at low wind speed. However, a positive association between wind speed and COVID-19 cases has also been reported in like New York, USA (Bashir et al. 2020), Oslo, Norway (Menebo 2020), and Turkey (Şahin 2020). Wind speed is of substantial importance in modulating the dynamics of pathogens and disease vectors (Ellwanger and Chies 2018). Considering the discrepancy in the findings related to the impact of environmental factors on the COVID-19 transmission rate, further investigations are required to get conclusive insights it.

Air quality has shown significant correlation with the extent of the COVID-19 cases. Initial investigations from China, Italy, and the Netherlands etc. provide compelling evidence that exposure to higher PM_2.5_ can result in increased morbidity, hospitalization, and fatality due to COVID-19 (Conticini et al. 2020; Fattorini and Regoli 2020; Wang et al. 2020a). A nationwide study in the USA suggested that air pollution has significantly worsened the outbreak and COVID-19-associated death counts (Wu et al. 2020). They suggested that an increase of just 1 μg/m^3^ in fine particulate matter (PM_2.5_ < 2.5 μm) corresponded to an 8% increase in the COVID-19 death rate. The evidence from SARS, the disease caused by another Corona virus, suggests that increased PM_2.5_ result in significantly higher mortality and morbidity (Comunian et al. 2020). They found that SARS patients residing in areas with high air pollution index were twice as likely to die from this disease than those from localities with lower air pollution. Significance of the air quality highlights the need to promote the green environmental policies.

#### COVID-19 and associated indirect environmental threats

During the outbreak, many types of additional environmental challenges have arisen such as exposure to disinfectants, increased production of solid waste, and reduced recycling, increased use of biomass, food insecurity, and halting of the projects for renewable energy projects. These challenges are briefly described below:

#### Increased exposure to the emerging environmental pollutants and disinfectants

Marinating proper hygienic conditions has been recognized as the first line of defense against COVID-19 which has increased the use of antibacterial soaps, household cleaning agents, and disinfectants (Gharpure et al. 2020; Usman et al. 2020a). Increased and sometimes unnecessary use of cleaners and disinfectants is also accompanied by the possibility of improper use such as using in more than directed on the label or mixing different products etc. (Gharpure et al. 2020). As biocides present in these products are not completely eliminated by the existing wastewater treatments (Kümmerer et al. 2018), these compounds may end up in water bodies and soil and thus may pose severe risks to the ecosystem. These compounds may also spread the antimicrobial resistance in the environment (Usman et al. 2020a). Similarly, disinfection routines (mainly relying on chlorination) at wastewater treatment plants are being strengthened to prevent the spread of this virus through wastewater (Zambrano-Monserrate et al. 2020).

##### Solid waste management

Generation of the medical waste is particularly on the rise. For example, in March 2020, Hubei Province (China) reported a sharp increase (by 370%) in the generation of medical waste with a high proportion of plastics (Klemeš et al. 2020). These face masks, being polymer-based, may serve as the source of microplastic fibers in the environment (Fadare and Okoffo 2020). Therefore, management of the huge amount of the medical waste and personal protective equipment has emerged as a new challenge during this pandemic (Fadare and Okoffo 2020; Peng et al. 2020; Rhee 2020). Since improper disposal of medical waste could be a source of infection, COVID-19-related waste should be dealt with strict implementation (Peng et al. 2020). Even before this pandemic, worldwide waste management systems were lagging behind particularly to address the plastic waste (Klemeš et al. 2020). During this pandemic, many incidents of improper transport, storage, and disposal of COVID-19-associated medical waste has been reported particularly in the developing countries (Mihai 2020; Times-of-India 2020). Similarly, use of disposable plastic products and packaging materials has been increased due to hygienic concerns contributing towards increased volume of solid waste (Klemeš et al. 2020). As highlighted by Klemeš et al. (2020), the amount of waste threatens to overwhelm existing treatment and disposal facilities. Problems with the solid waste management worsened because the recycling programs have been reduced during this pandemic due to the concerns of the spread of the COVID-19 at these facilities (Zambrano-Monserrate et al. 2020). It is worth mentioning that researchers have also been looking for suitable strategies to disinfect the face masks for reuse. For this, techniques like dry heat pasteurization (Xiang et al. 2020) and germicidal UV light (Zhao et al. 2020) were found satisfactory. On the other hand, autoclave sterilization and ethanol treatment affected the filtration efficiency and breathability of reused masks (Grinshpun et al. 2020). Researchers also highlighted the need to develop bio-based face masks to curb environmental pollution (Das et al. 2020).

##### Food insecurity

COVID-19 has a strong impact on crop production systems, livestock farming, supply chain, and global food availability (Fleetwood 2020; O’Kane 2020). Even the developed countries like Australia experienced pockets of food shortage (O’Kane 2020). Agriculture sector has suffered from the wastage of fresh fruits and vegetables, poultry, livestock, and dairy products due to restricted movements and the shortage of the workforce due to restricted movements and social distancing (World-Bank 2020). With families out of work and schools closed, food insecurity is expected to skyrocket (Kinsey et al. 2020). Researchers have also raised concerns that COVID-19 may threaten the implementation of the sustainable development goals (Fleetwood 2020; Leal Filho et al. 2020; Sakamoto et al. 2020). To address the problems of food supply, researchers have been proposing different strategies like urban agriculture (Pulighe and Lupia 2020), urban-rural collaborations (Cattivelli and Rusciano 2020; Sukhwani et al. 2020), shorter supply chains (Pulighe and Lupia 2020), and use of innovative agri-food systems based on artificial intelligence (Di Vaio et al. 2020). These studies highlight the need of alternative food systems having high resilience to such global crisis. It has also been suggested that combined efforts of volunteering association and local communities would be highly rewarding to respond to food provisioning problems during this pandemic (Cattivelli and Rusciano 2020).

#### Planning and management of cities and buildings

To better prepare the cities for future pandemics like COVID-19, researchers have been exploring different strategies for post-COVID-19 cities. During this crisis, inadequate health facilities and poor public services emerged as the crucial factors contributing towards low capacity to tackle this pandemic (Pisano 2020). For example, during this pandemic, Paris a concept of a “15 min city” in Paris which is intended to provide citizens’ basic needs, such as work, health, shopping, or culture, within 15 min of their home (Pisano 2020). Similarly, Milano 2020 has been proposed as an adaptation strategy in Milan (Italy) that also insists on revaluating the neighborhood size so that all necessary services are available within a walking distance (Pisano 2020). After a thorough investigation of these strategies, Pisano (2020) highlighted that following three factors in post-COVID-19 cities seem particularly relevant including (i) decentralization of facilities, (ii) hierarchization of the public services and transport system, and (iii) redundancy of public and semipublic functions. Similarly, Pineda and Corburn (2020) stressed that cities should be promoted to ensure equal access for all citizens, including those with disabilities through more inclusive community planning. Maintaining proper ventilation in buildings and good indoor air has also been regarded as an effective prevention strategy (Amoatey et al. 2020). Considering the risks associated contaminated air and sewage, researchers have also been highlighting the need to find better pollution control strategies in the buildings and cities as summarized in previous sections.

## Conclusion

Present bibliometric study has evaluated the publications related to the environment and the COVID-19 which are available in the Web of Science. Relevant articles were searched in 9 environment-related categories in Web of Science and after an initial evaluation, 495 articles were retained for further analysis.

Most of the articles were issued in journals like *Science of the Total Environment*, *Sustainability*, *International Journal of Environmental Research and Public Health*, and *Aerosol and Air Quality Research*. China, USA, and Italy were the leading countries in research related to this theme. We also identified the leading institutions working on this theme to facilitate the collaborations and other knowledge exchange activities.

Finally, this bibliometric study allowed visualization of the research trends that emerged prominently during this pandemic. It has been found that assessment of air and water quality has been widely focused. Sharp decline in air and water pollution have been reported due to COVID-19 imposed lockdowns. Concentration of major air pollutants like PM, NO_2_, and CO_2_ decreased except for O_3_ which increased in the air due to a pollution imbalance. Therefore, better understanding of complex chemistry of air pollutant formation, transport, and temporal variability is required. It was found that there exists a significant associations of air pollution in exacerbating COVID-19 which highlights the need of a clean environment in the time of a pandemic.

Similarly, existence of SARS-CoV-2 in sewage opened many avenues for an early monitoring of the prevalence of this disease in a community. However, researchers also raised concerns about the potential risks of COVID-19 transmission due to the exposure to this virus in sewage. Therefore, development and implementation of effective disinfection strategies for sewage treatment is needed. Environmental transmission of COVID-19 has been another important focus of research in this theme where effect of various meteorological factors has been studied. In addition to the direct threats associated with COVID-19, this pandemic has contributed in indirect threats like increased exposure to disinfectants and antimicrobials, food insecurity, and solid waste management. Disposal of infectious plastic waste has emerged as a prominent threat in many countries. Similarly, inadequate health facilities and poor public services emerged as the crucial factors hindering effective tackling of this pandemic. Therefore, researchers have also been stressing the need to learn from COVID-19 to ensure sustainable city planning and management in the event of future crisis.

## Data Availability

Data used in this study were retrieved from the Clarivate Analytics Web of Science Core Collection, the online version of the Science Citation Index Expanded (SCI-EXPANDED).

## References

[CR1] Ahmadi M, Sharifi A, Dorosti S, Ghoushchi SJ, Ghanbari N (2020). Investigation of effective climatology parameters on COVID-19 outbreak in Iran. Sci Total Environ.

[CR2] Ahmed W, Angel N, Edson J, Bibby K, Bivins A, O’Brien J, Choi PM, Kitajima M, Simpson SL, Li JY, Tscharke B, Verhagen R, Smithg WJM, Zaugg J, Dierens L, Hugenholtz P, Thomas KV, Mueller JF (2020). First confirmed detection of SARS-CoV-2 in untreated wastewater in Australia: a proof of concept for the wastewater surveillance of COVID-19 in the community. Sci Total Environ.

[CR3] Ahmed W, Bertsch PM, Bivins A, Bibby K, Farkas K, Gathercole A, Haramoto E, Gyawali P, Korajkic A, McMinn BR, Mueller JF, Simpson SL, Smith WJM, Symonds EM, Thomas KV, Verhagen R, Kitajima M (2020). Comparison of virus concentration methods for the RT-qPCR-based recovery of murine hepatitis virus, a surrogate for SARS-CoV-2 from untreated wastewater. Sci Total Environ.

[CR4] Amoatey P, Omidvarborna H, Baawain MS, Al-Mamun A (2020). Impact of building ventilation systems and habitual indoor incense burning on SARS-CoV-2 virus transmissions in Middle Eastern countries. Sci Total Environ.

[CR5] Balacco G, Totaro V, Iacobellis V, Manni A, Spagnoletta M, Piccinni AF (2020). Influence of COVID-19 spread on water drinking demand: the case of Puglia Region (Southern Italy). Sustainability.

[CR6] Bashir MF, Ma BJ, Bilal KB, Bashir MA, Tan DJ, Bashir M (2020). Correlation between climate indicators and COVID-19 pandemic in New York, USA. Sci Total Environ.

[CR7] Braga F, Scarpa GM, Brando VE, Manfe G, Zaggia L (2020). COVID-19 lockdown measures reveal human impact on water transparency in the Venice Lagoon. Sci Total Environ.

[CR8] Cahill N, Morris D (2020). Recreational waters: a potential transmission route for SARS-CoV-2 to humans?. Sci Total Environ.

[CR9] Cattivelli V, Rusciano V (2020). Social innovation and food provisioning during Covid-19: the case of urban-rural initiatives in the Province of Naples. Sustainability.

[CR10] Chiu WT, Ho YS (2005). Bibliometric analysis of homeopathy research during the period of 1991 to 2003. Scientometrics.

[CR11] Chiu WT, Ho YS (2007). Bibliometric analysis of tsunami research. Scientometrics.

[CR12] Colares GS, Dell’Osbel N, Wiesel PG, Oliveira GA, Lemos PHZ, da Silva FP, Lutterbeck CA, Kist LT, Machado EL (2020). Floating treatment wetlands: a review and bibliometric analysis. Sci Total Environ.

[CR13] Comunian S, Dongo D, Milani C, Palestini P (2020). Air pollution and COVID-19: the role of particulate matter in the spread and increase of COVID-19’s morbidity and mortality. Int J Environ Res Public Health.

[CR14] Conticini E, Frediani B, Caro D (2020). Can atmospheric pollution be considered a co-factor in extremely high level of SARS-CoV-2 lethality in Northern Italy?. Environ Pollut.

[CR15] Das O, Neisiany RE, Capezza AJ, Hedenqvist MS, Försth M, Xu Q, Jiang L, Ji DX, Ramakrishna S (2020). The need for fully bio-based facemasks to counter coronavirus outbreaks: a perspective. Sci Total Environ.

[CR16] Di Vaio A, Boccia F, Landriani L, Palladino R (2020). Artificial intelligence in the agri-food system: rethinking sustainable business models in the COVID-19 scenario. Sustainability.

[CR17] Ellwanger JH, Chies JAB (2018). Wind: A neglected factor in the spread of infectious diseases. Lancet Planet Health.

[CR18] Fadare OO, Okoffo ED (2020). Covid-19 face masks: a potential source of microplastic fibers in the environment. Sci Total Environ.

[CR19] Fattorini D, Regoli F (2020). Role of the chronic air pollution levels in the Covid-19 outbreak risk in Italy. Environ Pollut.

[CR20] Fleetwood J (2020). Social justice, food loss, and the sustainable development goals in the era of COVID-19. Sustainability.

[CR21] Gharpure R, Hunter CM, Schnall AH, Barrett CE, Kirby AE, Kunz J, Berling K, Mercante JW, Murphy JL, Garcia-Williams AG (2020). Knowledge and practices regarding safe household cleaning and disinfection for COVID-19 prevention - United States, May 2020. Morb Mortal Wkly Rep.

[CR22] Grinshpun SA, Yermakov M, Khodoun M (2020). Autoclave sterilization and ethanol treatment of re-used surgical masks and N95 respirators during COVID-19: impact on their performance and integrity. J Hosp Infect.

[CR23] Haramoto E, Malla B, Thakali O, Kitajima M (2020). First environmental surveillance for the presence of SARS-CoV-2 RNA in wastewater and river water in Japan. Sci Total Environ.

[CR24] Hart OE, Halden RU (2020). Computational analysis of SARS-CoV-2/COVID-19 surveillance by wastewater-based epidemiology locally and globally: feasibility, economy, opportunities and challenges. Sci Total Environ.

[CR25] Hsu YHE, Ho YS (2014). Highly cited articles in health care sciences and services field in Science Citation Index Expanded: a bibliometric analysis for 1958-2012. Methods Inf Med.

[CR26] Jahangiri M, Jahangiri M, Najafgholipour M (2020). The sensitivity and specificity analyses of ambient temperature and population size on the transmission rate of the novel coronavirus (COVID-19) in different provinces of Iran. Sci Total Environ.

[CR27] Kinsey EW, Kinsey D, Rundle AG (2020). COVID-19 and food insecurity: an uneven patchwork of responses. J. Urban Health.

[CR28] Klemeš JJ, Van Fan Y, Tan RR, Jiang P (2020). Minimising the present and future plastic waste, energy and environmental footprints related to COVID-19. Renew Sust Energ Rev.

[CR29] Kümmerer K, Dionysiou DD, Olsson O, Fatta-Kassinos D (2018). A path to clean water. Science.

[CR30] La Rosa G, Iaconelli M, Mancini P, Ferraro GB, Veneri C, Bonadonna L, Lucentini L, Suffredini E (2020). First detection of SARS-CoV-2 in untreated wastewaters in Italy. Sci Total Environ.

[CR31] Leal Filho W, Brandli LL, Salvia AL, Rayman-Bacchus L, Platje J (2020). COVID-19 and the UN sustainable development goals: threat to solidarity or an opportunity?. Sustainability.

[CR32] Li Z, Ho YS (2008). Use of citation per publication as an indicator to evaluate contingent valuation research. Scientometrics.

[CR33] Li JF, Zhang YH, Wang XS, Ho YS (2009). Bibliometric analysis of atmospheric simulation trends in meteorology and atmospheric science journals. Croat Chem Acta.

[CR34] Lin CQ, Lau AKH, Fung JCH, Guo C, Chan JWM, Yeung DW, Zhang YM, Bo YC, Hossain MS, Zeng YQ, Lao XQ (2020). A mechanism-based parameterisation scheme to investigate the association between transmission rate of COVID-19 and meteorological factors on plains in China. Sci Total Environ.

[CR35] Lodder W, Husman AMD (2020). SARS-CoV-2 in wastewater: potential health risk, but also data source. Lancet Gastroenterol Hepatol.

[CR36] Mandal I, Pal S (2020). COVID-19 pandemic persuaded lockdown effects on environment over stone and areas. Sci Total Environ.

[CR37] McDermott CV, Alicic RZ, Harden N, Cox EJ, Scanlan JM (2020). Put a lid on it: are faecal bio-aerosols a route of transmission for SARS-CoV-2?. J Hosp Infect.

[CR38] Menebo MM (2020). Temperature and precipitation associate with Covid-19 new daily cases: a correlation study between weather and Covid-19 pandemic in Oslo, Norway. Sci Total Environ.

[CR39] Mihai FC (2020). Assessment of COVID-19 waste flows during the emergency state in Romania and related public health and environmental concerns. Int J Environ Res Public Health.

[CR40] Mlejnkova H, Sovova K, Vasickova P, Ocenaskova V, Jasikova L, Juranova E (2020). Preliminary study of SARS-CoV-2 occurrence in wastewater in the Czech Republic. Int J Environ Res Public Health.

[CR41] Morawska L, Cao JJ (2020). Airborne transmission of SARS-CoV-2: The world should face the reality. Environ Int.

[CR42] Muhammad S, Long XL, Salman M (2020). COVID-19 pandemic and environmental pollution: a blessing in disguise?. Sci Total Environ.

[CR43] O’Kane G (2020). COVID-19 puts the spotlight on food insecurity in rural and remote Australia. Aust J Rural Health.

[CR44] Pani SK, Lin NH, RavindraBabu S (2020) Association of COVID-19 pandemic with meteorological parameters over Singapore. Sci Total Environ 740:140112. 10.1016/j.scitotenv.2020.140112 10.1016/j.scitotenv.2020.140112 PMC7289735 32544735

[CR45] Peng J, Wu XL, Wang RL, Li C, Zhang Q, Wei DQ (2020). Medical waste management practice during the 2019-2020 novel coronavirus pandemic: experience in a general hospital. Am J Infect Control.

[CR46] Pineda VS, Corburn J (2020). Disability, urban health equity, and the coronavirus pandemic: promoting cities for all. J Urban Health.

[CR47] Pisano C (2020). Strategies for post-COVID cities: an insight to Paris en Commun and Milano 2020. Sustainability.

[CR48] Prata DN, Rodrigues W, Bermejo PH (2020). Temperature significantly changes COVID-19 transmission in (sub) tropical cities of Brazil. Sci Total Environ.

[CR49] Pulighe G, Lupia F (2020). Food first: COVID-19 outbreak and cities lockdown a booster for a wider vision on urban agriculture. Sustainability.

[CR50] Randazzo W, Truchado P, Cuevas-Ferrando E, Simón P, Allende A, Sánchez G (2020). SARS-CoV-2 RNA in wastewater anticipated COVID-19 occurrence in a low prevalence area. Water Res.

[CR51] Rhee SW (2020). Management of used personal protective equipment and wastes related to COVID-19 in South Korea. Waste Manag Res.

[CR52] Romano-Bertrand S, Glele LSA, Grandbastien B, Lepelletier D (2020). Preventing SARS-CoV-2 transmission in rehabilitation and water environments. J Hosp Infect.

[CR53] Şahin M (2020). Impact of weather on COVID-19 pandemic in Turkey. Sci Total Environ.

[CR54] Sakamoto M, Begum S, Ahmed T (2020). Vulnerabilities to COVID-19 in Bangladesh and a reconsideration of sustainable development goals. Sustainability.

[CR55] Selvam S, Jesuraja K, Venkatramanan S, Chung SY, Roy PD, Muthukumar P, Kumar M (2020). Imprints of pandemic lockdown on subsurface water quality in the coastal industrial city of Tuticorin, South India: a revival perspective. Sci Total Environ.

[CR56] Sharifi A, Khavarian-Garmsir AR (2020). The COVID-19 pandemic: impacts on cities and major lessons for urban planning, design, and management. Sci Total Environ.

[CR57] Sharma S, Zhang MY, Anshika GJS, Zhang HL, Kota SH (2020). Effect of restricted emissions during COVID-19 on air quality in India. Sci Total Environ.

[CR58] Sicard P, De Marco A, Agathokleous E, Feng ZZ, Xu XB, Paoletti E, Rodriguez JJD, Calatayud V (2020). Amplified ozone pollution in cities during the COVID-19 lockdown. Sci Total Environ.

[CR59] Sobral MFF, Duarte GB, Sobral AIGD, Marinho MLM, Melo AD (2020). Association between climate variables and global transmission of SARS-CoV-2. Sci Total Environ.

[CR60] Somala SN (2020). Seismic noise changes during COVID-19 pandemic: a case study of Shillong, India. Nat Hazards.

[CR61] Sukhwani V, Deshkar S, Shaw R (2020). COVID-19 lockdown, food systems and urban-rural partnership: case of Nagpur, India. Int J Environ Res Public Health.

[CR62] Times-of-India (2020) Chennai: biomedical waste found near Chembarambakkam lake again. https://timesofindia.indiatimes.com/city/chennai/chennai-biomedical-waste-found-near-chembarambakkam-lake-again/articleshow/79472254.cms (accessed on December 02, 2020)

[CR63] Usman M, Ho YS (2020). A bibliometric study of the Fenton oxidation for soil and water remediation. J Environ Manag.

[CR64] Usman M, Farooq M, Hanna K (2020). Environmental side effects of the injudicious use of antimicrobials in the era of COVID-19. Sci Total Environ.

[CR65] Usman M, Farooq M, Hanna K (2020). Existence of SARS-CoV-2 in wastewater: implications for its environmental transmission in developing communities. Environ Sci Technol.

[CR66] Vanzetto GV, Thomé A (2019). Bibliometric study of the toxicology of nanoescale zero valent iron used in soil remediation. Environ Pollut.

[CR67] Wang CC, Ho YS (2016). Research trend of metal-organic frameworks: a bibliometric analysis. Scientometrics.

[CR68] Wang WL, Yoneda M (2020). Determination of the optimal penetration factor for evaluating the invasion process of aerosols from a confined source space to an uncontaminated area. Sci Total Environ.

[CR69] Wang BM, Chen H, Chan YL, Oliver BG (2020). Is there an association between the level of ambient air pollution and COVID-19?. Am J Physiol-Lung Cell Mol Physiol.

[CR70] Wang LQ, Li MY, Yu SC, Chen X, Li Z, Zhang YB, Jiang LH, Xia Y, Li JL, Liu WP, Li PF, Lichtfouse E, Rosenfeld D, Seinfeld JH (2020). Unexpected rise of ozone in urban and rural areas, and sulfur dioxide in rural areas during the coronavirus city lockdown in Hangzhou, China: implications for air quality. Environ Chem Lett.

[CR71] Wang PF, Chen KY, Zhu SQ, Wang P, Zhang HL (2020). Severe air pollution events not avoided by reduced anthropogenic activities during COVID-19 outbreak. Resour Conserv Recycl.

[CR72] WHO (2020) Modes of transmission of virus causing COVID-19: implications for IPC precaution recommendations. https://www.who.int/news-room/commentaries/detail/transmission-of-SARS-CoV-2-implications-for-infection-prevention-precautions. Accessed 10 Aug 2020

[CR73] World-Bank. Food Security and COVID-19. https://www.worldbank.org/en/topic/agriculture/brief/food-security-and-covid-19 (accessed on August 07, 2020), 2020.

[CR74] Worldometers (2020) Coronavirus updates. https://www.worldometers.info/coronavirus/ (Accessed on August 14, 2020)

[CR75] Wu X, Nethery RC, Sabath MB, Braun D, Dominici F (2020). Air pollution and COVID-19 mortality in the United States: strengths and limitations of an ecological regression analysis. Sci Adv.

[CR76] Xiang Y, Song QF, Gu WZ (2020). Decontamination of surgical face masks and N95 respirators by dry heat pasteurization for one hour at 70°C. Am J Infect Control.

[CR77] Xu H, Yan CH, Fu QY, Xiao K, Yu YM, Han DM, Wang WH, Cheng JP (2020). Possible environmental effects on the spread of COVID-19 in China. Sci Total Environ.

[CR78] Yunus AP, Masago Y, Hijioka Y (2020). COVID-19 and surface water quality: improved lake water quality during the lockdown. Sci Total Environ.

[CR79] Zambrano-Monserrate MA, Ruano MA, Sanchez-Alcalde L (2020). Indirect effects of COVID-19 on the environment. Sci Total Environ.

[CR80] Zhao Z, Zhang ZB, Lanzarini-Lopes M, Sinha S, Rho HJ, Herckes P, Westerhoff P (2020). Germicidal ultraviolet light does not damage or impede performance of n95 masks upon multiple uses. Environ Sci Technol Lett.

[CR81] Zoran MA, Savastru RS, Savastru DM, Tautan MN (2020). Assessing the relationship between ground levels of ozone (O_3_) and nitrogen dioxide (NO_2_) with coronavirus (COVID-19) in Milan, Italy. Sci Total Environ.

[CR82] Zoran MA, Savastru RS, Savastru DM, Tautan MN (2020). Assessing the relationship between surface levels of PM_2.5_ and PM_10_ particulate matter impact on COVID-19 in Milan, Italy. Sci Total Environ.

